# Humans Surviving Cholera Develop Antibodies against Vibrio cholerae O-Specific Polysaccharide That Inhibit Pathogen Motility

**DOI:** 10.1128/mBio.02847-20

**Published:** 2020-11-17

**Authors:** Richelle C. Charles, Meagan Kelly, Jenny M. Tam, Aklima Akter, Motaher Hossain, Kamrul Islam, Rajib Biswas, Mohammad Kamruzzaman, Fahima Chowdhury, Ashraful I. Khan, Daniel T. Leung, Ana Weil, Regina C. LaRocque, Taufiqur Rahman Bhuiyan, Atiqur Rahman, Leslie M. Mayo-Smith, Rachel L. Becker, Jatin M. Vyas, Christina S. Faherty, Kourtney P. Nickerson, Samantha Giffen, Alaina S. Ritter, Matthew K. Waldor, Peng Xu, Pavol Kováč, Stephen B. Calderwood, Robert C. Kauffman, Jens Wrammert, Firdausi Qadri, Jason B. Harris, Edward T. Ryan

**Affiliations:** a Division of Infectious Diseases, Massachusetts General Hospital, Boston, Massachusetts, USA; b Department of Medicine, Harvard Medical School, Boston, Massachusetts, USA; c International Centre for Diarrhoeal Disease Research, Bangladesh, Dhaka, Bangladesh; d Division of Infectious Diseases, Department of Internal Medicine, University of Utah School of Medicine, Salt Lake City, Utah, USA; e Department of Pediatrics, MassGeneral Hospital for Children, Boston, Massachusetts, USA; f Mucosal Immunology and Biology Research Center, Division of Pediatric Gastroenterology and Nutrition, Massachusetts General Hospital, Boston, Massachusetts, USA; g Department of Immunology and Infectious Diseases, Harvard T.H. Chan School of Public Health, Boston, Massachusetts, USA; h Department of Microbiology, Harvard Medical School, Boston, Massachusetts, USA; i Division of Infectious Diseases, Brigham and Women’s Hospital, Boston, Massachusetts, USA; j Howard Hughes Medical Institute, Boston, Massachusetts, USA; k NIDDK, LBC, National Institutes of Health, Bethesda, Maryland, USA; l Division of Infectious Disease, Department of Pediatrics, Emory University School of Medicine, Atlanta, Georgia, USA; m Emory Vaccine Center, Emory University School of Medicine, Atlanta, Georgia, USA; n Division of Pediatric Global Health, Massachusetts General Hospital, Boston, Massachusetts, USA; Fred Hutchinson Cancer Research Center

**Keywords:** *Vibrio cholerae*, cholera, human, motility, pathogenesis

## Abstract

The mechanism of protection against cholera afforded by previous illness or vaccination is currently unknown. We have recently shown that antibodies targeting O-specific polysaccharide (OSP) of Vibrio cholerae correlate highly with protection against cholera. V. cholerae is highly motile and possesses a flagellum sheathed in OSP, and motility of V. cholerae correlates with virulence. Using high-speed video microscopy and building upon previous animal-related work, we demonstrate that sera, polyclonal antibody fractions, and OSP-specific monoclonal antibodies recovered from humans surviving cholera block V. cholerae motility at both subagglutinating and agglutinating concentrations. This antimotility effect is reversed by preadsorbing sera and polyclonal antibody fractions with purified OSP and is associated with OSP-specific but not flagellin-specific monoclonal antibodies. Fab fragments of OSP-specific polyclonal antibodies do not inhibit motility, suggesting a requirement for antibody-mediated cross-linking in motility inhibition. We show that OSP-specific antibodies do not directly affect V. cholerae viability, but that OSP-specific monoclonal antibody highly protects against death in the murine cholera model. We used *in vivo* competitive index studies to demonstrate that OSP-specific antibodies impede colonization and survival of V. cholerae in intestinal tissues and that this impact is motility dependent. Our findings suggest that the impedance of motility by antibodies targeting V. cholerae OSP contributes to protection against cholera.

## Abstract (precis)

Cholera is a severe dehydrating illness of humans caused by Vibrio cholerae. V. cholerae is a highly motile bacterium that has a single flagellum covered in lipopolysaccharide (LPS) displaying O-specific polysaccharide (OSP), and V. cholerae motility correlates with its ability to cause disease. The mechanisms of protection against cholera are not well understood; however, since V. cholerae is a noninvasive intestinal pathogen, it is likely that antibodies that bind the pathogen or its products in the intestinal lumen contribute to protection from infection. Here, we demonstrate that OSP-specific antibodies isolated from humans surviving cholera in Bangladesh inhibit V. cholerae motility and are associated with protection against challenge in a motility-dependent manner.

## INTRODUCTION

Cholera is a severe dehydrating illness of humans caused almost exclusively by Vibrio cholerae of the O1 serogroup. Over 1 billion people remain at risk for cholera in 51 countries of endemicity, and there are an estimated 3 million cases and 95,000 deaths each year from cholera (1). The current global pandemic began in 1961 and gives no indication of abating, as evidenced by recent large outbreaks in Haiti and Yemen (2). This reality has led to enhanced commitments to cholera control strategies (3). Such strategies now include vaccination against cholera, along with efforts to improve water and sanitation (4). Currently available oral killed cholera vaccines are an important addition to these control efforts; however, these vaccines may provide limited durable protection, especially in immunologically naive individuals, including children under 5 years of age who bear a large proportion of the global cholera burden (2). In comparison, survivors of cholera, including young children, have high-level protective immunity that persists for years (5).

The development and optimal use of cholera vaccines has been hampered by the relatively limited understanding of the immunologic mechanisms of protection against cholera. V. cholerae is a noninvasive luminal intestinal pathogen, and it is likely that antibodies that bind the pathogen or its products in the intestinal lumen contribute to protective immunity (6). Cholera is a toxin-mediated disease; V. cholerae express cholera toxin (CT), an ADP-ribosylating enzyme, at the intestinal surface, and the actions of this toxin on intestinal epithelial cells lead to the large-volume secretory diarrhea characteristic of cholera (7). Despite this, immune responses that target CT do not provide meaningful protection against cholera (8).

An *in vitro* vibriocidal assay is currently our best predictor of protection against cholera; however, the vibriocidal response appears to be a surrogate marker of an as-yet-to-be-identified mucosal antibody response(s) (7). We have shown that the vibriocidal response largely targets the O-specific polysaccharide (OSP) of V. cholerae (9). Moreover, we found that OSP-specific antibody and memory B cell responses correlate with protection against cholera in household contacts of cholera index patients in Bangladesh (10). In North American recipients of an oral cholera vaccine, OSP-specific antibody responses correlate with protection against cholera in challenge studies (11).

How OSP-specific antibodies protect against V. cholerae in the intestinal lumen is currently unclear. Possible mechanisms include direct bactericidal, enchaining, or agglutinating activity (12). However, we hypothesized that inhibition of motility could be a potential mechanism as well, a possibility supported by previous work (13–16). V. cholerae is a highly motile bacterium that has a single polar flagellum sheathed in lipopolysaccharide (LPS) displaying OSP, and V. cholerae motility correlates with virulence (17–19). Furthermore, several *in vitro* studies have shown that antibodies targeting V. cholerae LPS impede V. cholerae motility (13–16), and studies in suckling mice have suggested that the impedance of motility by anti-OSP antibodies provides protection in this model (13, 14, 16, 20, 21). Here, we used antibodies recovered from humans surviving cholera in Bangladesh, including monoclonal antibodies cloned from plasmablasts homing to the intestinal mucosa (22), to investigate the role of anti-OSP antibodies in protection against cholera. Our findings suggest that the inhibition of motility by the bivalent binding of anti-OSP antibodies contributes to immune protection from cholera.

## RESULTS

### Polyclonal OSP-specific antibodies inhibit V. cholerae motility.

**FIG 1:**
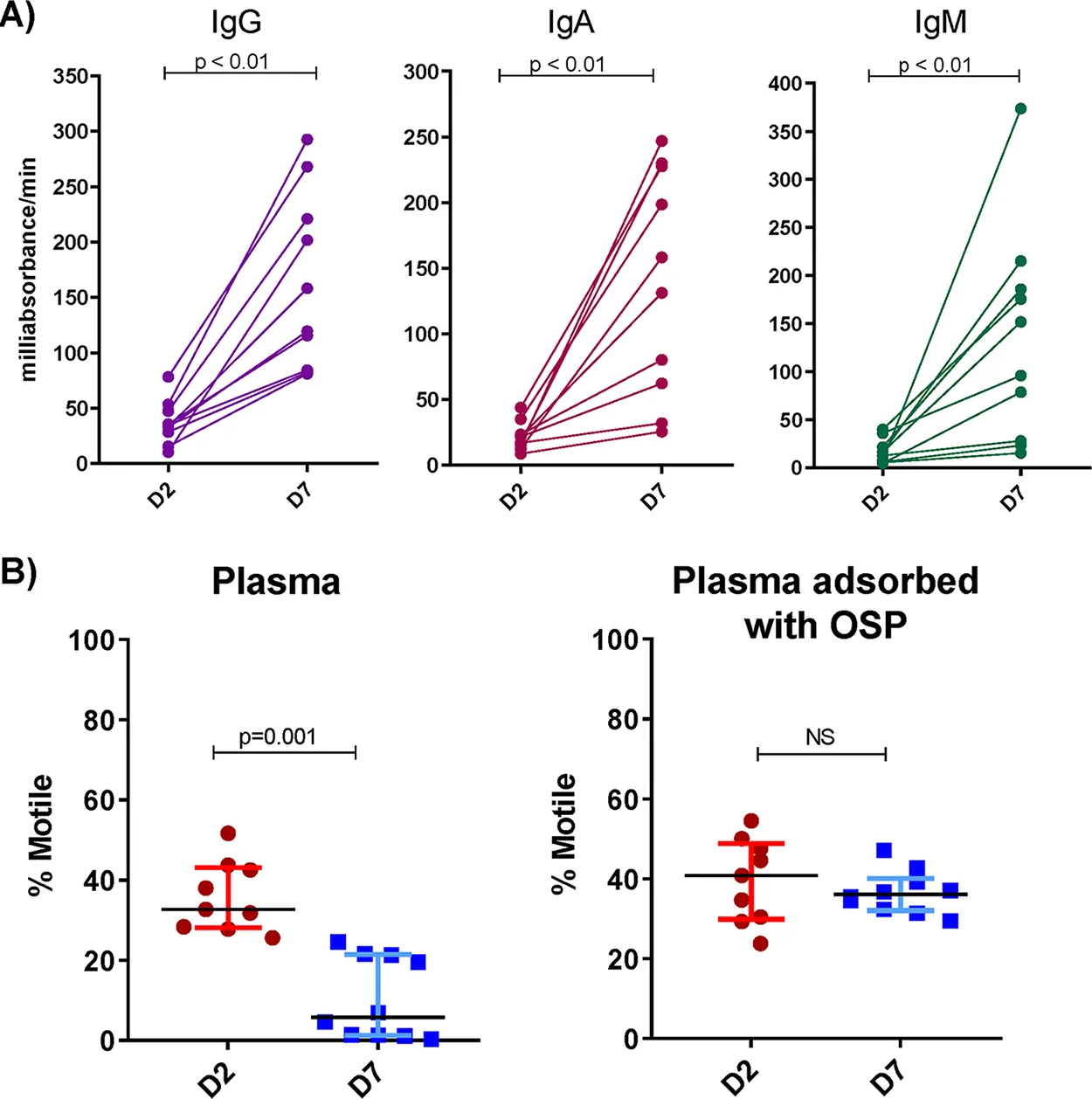
Convalescent-phase plasma of cholera patients recognizes V. cholerae OSP and inhibits V. cholerae motility in an OSP-dependent manner. (A) IgG, IgA, and IgM plasma responses targeting V. cholerae O1 Ogawa OSP at the acute (D2) and convalescent (D7) phase of cholera (*n* = 10) as determined by enzyme-linked immunosorbent assay. (B) Motility of V. cholerae O1 Ogawa O395 assessed by high-speed video microscopy after a 5-min incubation with a subagglutinating dilution of plasma (1:256) of cholera patients (*n* = 10) at a bacterial OD_600_ of 0.1. Percentages of motile versus nonmotile bacteria after incubation with heat-inactivated plasma with and without adsorption with V. cholerae OSP are shown. Bars show the medians with interquartile ranges. Differences within groups were assessed using the Wilcoxon matched-pairs signed-rank sums test. D2, day 2; D7, day 7; OSP, O-specific polysaccharide.

We analyzed polyclonal antibody responses in plasma from 10 adults recovering from cholera caused by V. cholerae O1 Ogawa in Bangladesh (see Table S1 in the supplemental material). These patients all developed IgG, IgA, and IgM responses against V. cholerae O1 OSP (*P* < 0.01 for all antibody isotypes) (Fig. 1A). The anti-V. cholerae O1 Ogawa agglutination activity was present in plasma across a range of dilutions in individual patients (1:64 to >1:8) (Table 1). Using high-speed video dark-field microscopy, we then assessed motility after incubating V. cholerae O1 with acute and convalescent-phase heat-inactivated plasma at subagglutinating concentrations (1:256) under the same conditions used for the agglutination assays. There was a significant decrease in motility when V. cholerae organisms were mixed with convalescent plasma compared to that with acute plasma (*P* = 0.001) (Fig. 1B). Adsorbing plasma with purified V. cholerae OSP eliminated its capacity to inhibit V. cholerae motility (Fig. 1B).

**TABLE 1:** V. cholerae O1 Ogawa agglutination activity of antibody samples

Sample	Day 7 value	
	Plasma titer^*a*^	IgG (μM)^*b*^
Patient no.		
1	8	>1
2	16	>1
3	<8	>0.5
4	<8	>1
5	<8	>2.5
6	8	>2.5
7	16	>1
8	64	>1
9	8	>1
10	<8	>1
Monoclonal antibody^*c*^		
Anti-OSP G1 (CF21.2.G01)		>2.048
Anti-OSP A4 (CF21.1.A04)		>2.048
Anti-OSP B4 (CF21.1.B04)		1.024
Anti-FlaA-flagellin AT11 (AT11.1.B12)		>2.048

a Plasma titers are reciprocal of last 2-fold dilution associated with agglutination using a multiwell system (see text). Starting dilution was 1:8; if no agglutination was evident at that dilution, the plasma result was marked as <8.

b Purified IgG fraction agglutination was checked via serial 2-fold dilutions beginning at 2.5, 1, or 0.5 μM depending on sample availability. No agglutination was noted in this assessment of purified polyclonal IgG, and the results are expressed as “>” the maximal starting dilution. Fab monomers of IgG were also checked for agglutinating activity via serial dilution beginning at 1 μM. No agglutinating activity was noted for any Fab sample.

c Monoclonal IgG antibodies were assessed for agglutinating activity via 2-fold dilutions beginning at 2.048 μM (0.320 mg/ml) (22). If no agglutination was evident at that dilution, the plasma result was marked as >2.048 μM. OSP, O-specific polysaccharide.

10.1128/mBio.02847-20.4 TABLE S1 Cholera patient characteristics for polyclonal sera. Download Table S1, DOCX file, 0.1 MB. Copyright © 2020 Charles et al. 2020 Charles et al. This content is distributed under the terms of the Creative Commons Attribution 4.0 International license.

**FIG 2:**
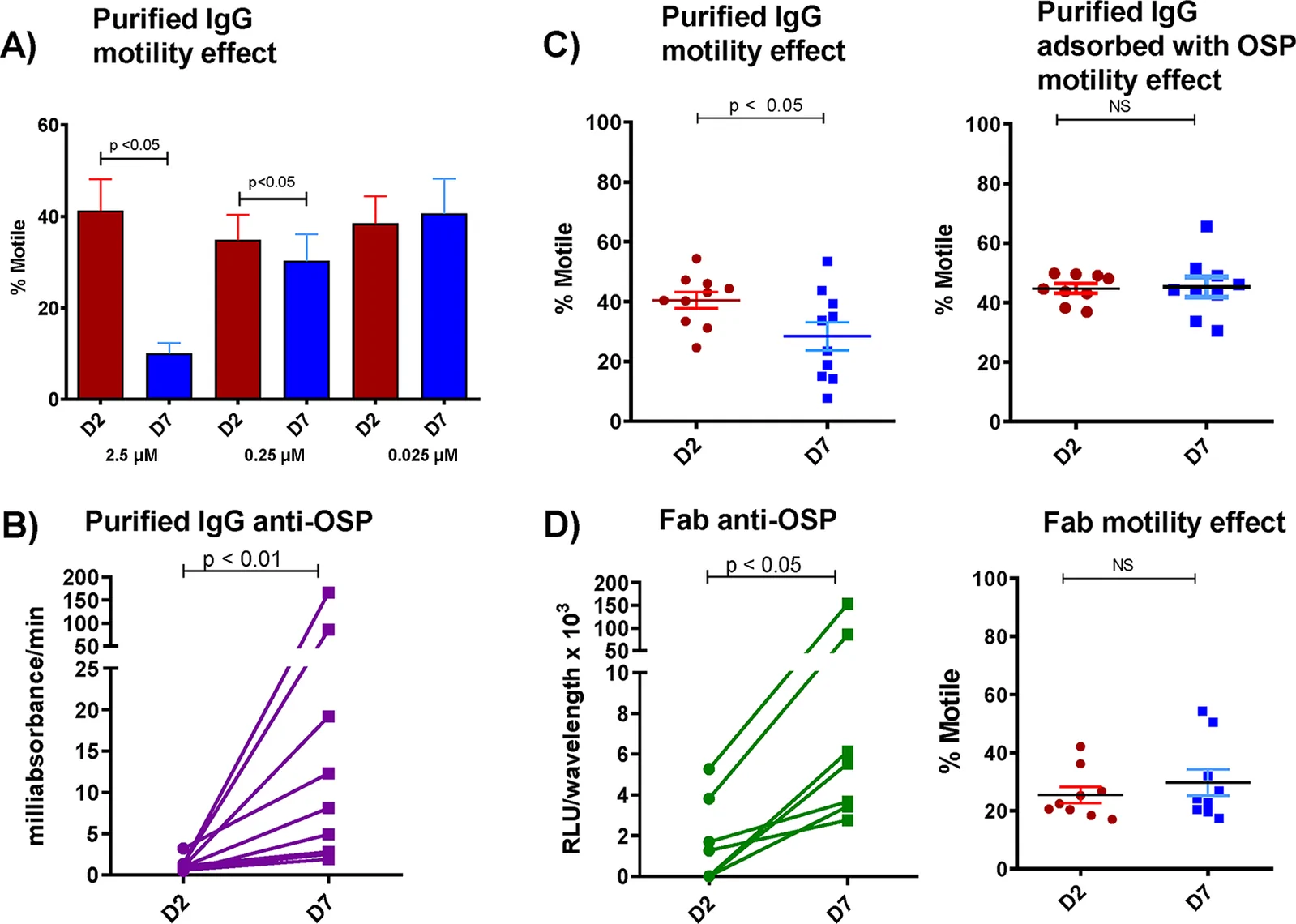
Purified IgG from humans recovering from cholera inhibits V. cholerae motility in a concentration-dependent manner, and this effect is eliminated when IgG is adsorbed with V. cholerae OSP. (A) IgG purified from pooled convalescent-phase plasma of humans recovering from cholera inhibits V. cholerae motility in a concentration-dependent manner. (B) OSP-specific responses of purified IgG from individual patients measured by enzyme-linked immunofluorescence assay showing day-7 immunoreactivity. (C) Purified polyclonal IgG at a subagglutinating 0.25 μM concentration from humans recovering from cholera inhibits V. cholerae motility, and this effect is eliminated when IgG is adsorbed with V. cholerae OSP. (D) Fab fragments generated from purified IgG retain day-7 immunoreactivity to OSP but lose ability to inhibit V. cholerae motility. D2, day 2; D7, day 7. Differences within groups were assessed using the Wilcoxon matched-pairs signed-rank sums test.

We next isolated IgG, IgA, and IgM antibody fractions from these plasma samples and focused our analyses on the IgG fraction, the most abundant isotype recovered. Using conditions that replicated those used in the video microscopy, we did not detect agglutination in 2-fold dilutions of purified IgG starting at 0.5, 1, or 2.5 μM depending on sample availability (Table 1). Starting at a concentration of 2.5 μM, we found the impact of pooled IgG on V. cholerae motility was concentration dependent and detectable at a subagglutinating concentration of 0.25 μM (Fig. 2A) (*P* ≤ 0.05) and independent of effects on V. cholerae viability (see Fig. S1). Purified IgG fractions from individual patients had similar anti-OSP reactivity (Fig. 2B) (*P* ≤ 0.01), and inhibition of V. cholerae motility at 0.25 μM was abrogated by adsorbing the IgG with purified V. cholerae OSP (Fig. 2C) (*P* ≤ 0.05). The absence of bacterial clumping/agglutination at 0.25 μM IgG was confirmed by microscopy. Thus, polyclonal IgG anti-OSP antibodies in convalescent patient sera inhibit the cholera pathogen’s motility at concentrations that do not cause the organism to agglutinate.

10.1128/mBio.02847-20.1 FIG S1 Viability of V. cholerae is not affected by plasma or purified IgG in absence of complement. Wild-type V. cholerae classical strain O395 (OD of 0.1) was mixed 1:1 with heat-inactivated convalescent-phase plasma (diluted 1:256) or purified IgG (0.25 μM and 2.5 μM) and incubated for 5 min before serial dilutional plating on LB agar. Each symbol represents the CFU/ml for a single experiment. Bars represent medians with interquartile ranges. Differences between groups were assessed using a Mann-Whitney U test. A *P* value smaller than 0.05 was considered significant. Download FIG S1, TIF file, 0.1 MB. Copyright © 2020 Charles et al. 2020 Charles et al. This content is distributed under the terms of the Creative Commons Attribution 4.0 International license.

To assess whether antibody-based cross-linking was required for inhibition of V. cholerae motility, we generated fragment antigen-binding (Fab) fragments from the IgG fractions of individual patients. In contrast to intact IgG antibodies, which both bound V. cholerae OSP (Fig. 2B) (*P* < 0.01) and blocked V. cholerae motility (Fig. 2C) (*P* ≤ 0.05), Fab fragments bound OSP (Fig. 2D) (*P* ≤ 0.01) but did not inhibit motility (Fig. 2D), suggesting that multivalent binding of OSP by antibody molecules is important for impeding motility. Supporting this idea, we found that dimeric IgA and polymeric IgM fractions also inhibited V. cholerae motility in an OSP-dependent fashion (see Fig. S2) (*P* ≤ 0.001).

10.1128/mBio.02847-20.2 FIG S2 Inhibition of V. cholerae O1 O395 motility by pooled purified IgA and IgM plasma fractions of humans recovering from cholera in Bangladesh. Pooled IgA (0.25 μM) and IgM (0.25 μM) fractions of convalescent-phase plasma of cholera patients (*N* = 10) also block V. cholerae (OD of 0.1) motility, and this effect is reduced by adsorption with V. cholerae OSP. d2, day 2; d7, day 7; d7 ads, day 7 adsorbed with OSP. Download FIG S2, TIF file, 0.1 MB. Copyright © 2020 Charles et al. 2020 Charles et al. This content is distributed under the terms of the Creative Commons Attribution 4.0 International license.

### Monoclonal OSP-specific antibodies inhibit V. cholerae motility.

**FIG 3:**
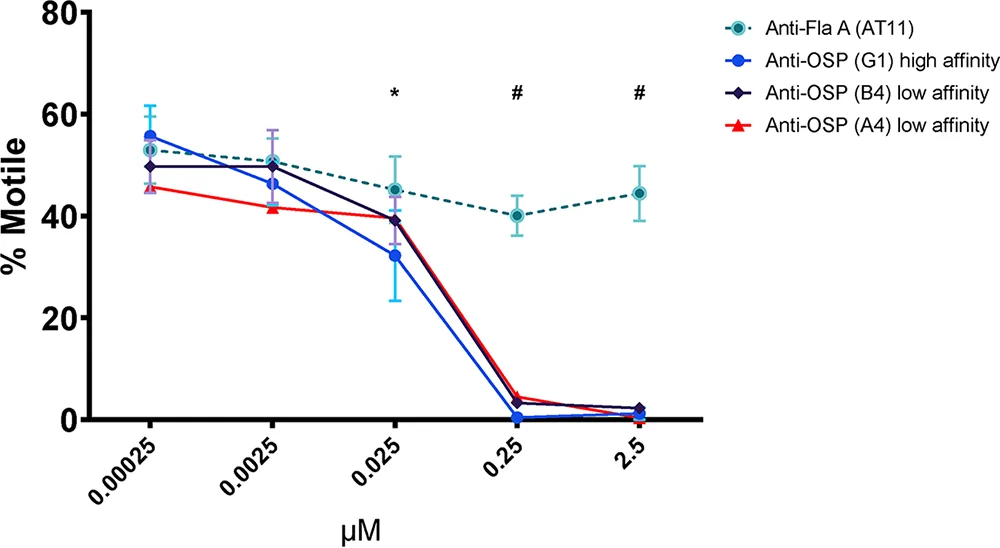
Human monoclonal antibodies targeting V. cholerae OSP inhibit motility in a concentration-dependent manner. Motility of V. cholerae O1 (OD_600_ of 0.1) was measured by phase contrast video microscopy after a 5-min incubation with increasing concentrations of human monoclonal IgG antibodies targeting OSP (high and low affinity) or flagellin A (FlaA). Each point represents the mean from experiments performed in triplicates. Error bars are the standard errors of the mean. *, *P* ≤ 0.05 G1 compared to 0.00025 μM; #, *P* ≤ 0.001 G1, A4, and B4 compared to 0.00025 μM.

**FIG 4:**
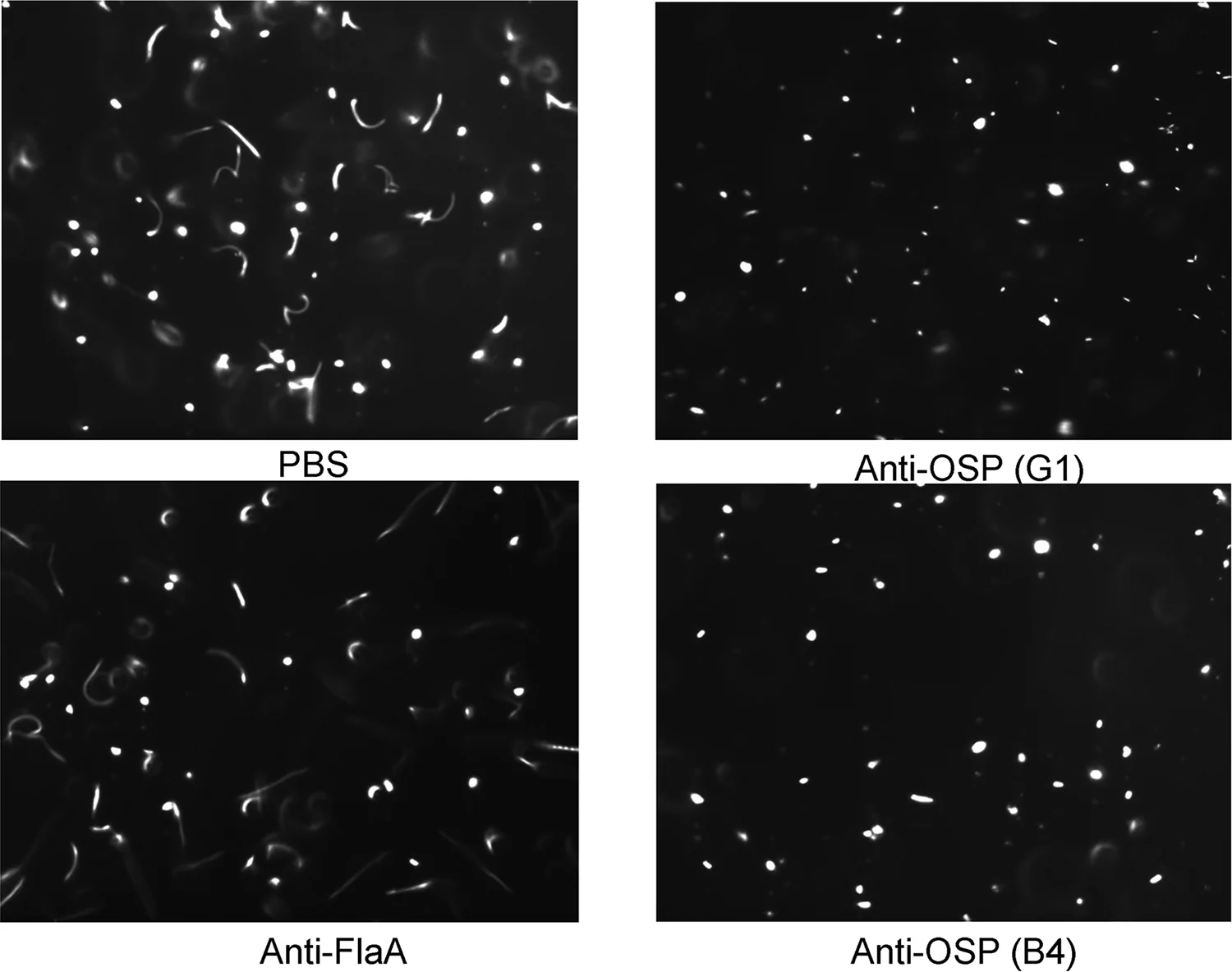
Freeze-frame phase contrast demonstrating anti-OSP human monoclonal antibodies eliminate V. cholerae shooting star motility, while anti-flagellin monoclonal antibody does not. Freeze frame (400 ms) images of V. cholerae O1 Ogawa O395 (OD of 0.1) after a 5-min incubation with phosphate-buffered saline or with human monoclonal antibodies (0.25 μM) targeting OSP (G1 and B4) or FlaA. Lines represent motile bacteria (shooting star); white dots represent nonmotile bacteria. Images were taken from high-speed videos and are representative of experiments performed in triplicates. Note elimination of white streaks demonstrating shooting star V. cholerae motility in the presence of OSP-specific monoclonal antibodies.

We next turned our attention to analyses of V. cholerae-specific monoclonal antibodies isolated from intestinal mucosa-homing plasmablasts from the peripheral blood of cholera patients sampled 7 days after they presented for clinical care in Bangladesh (22). We previously cloned (as human IgG1) and characterized >100 monoclonal antibodies from this collection (22). For the present analysis, we selected 4 previously characterized antibodies, including one monoclonal antibody with high affinity for V. cholerae OSP (G1), two antibodies with low affinity for V. cholerae OSP (A4 and B4), and one specific for V. cholerae flagellin A (AT11) (22). The OSP-specific monoclonal IgG antibodies were previously shown to recognize both Ogawa and Inaba antigens (22). Monoclonal IgG antibodies were assessed for agglutinating activity via 2-fold dilutions beginning at 2.048 μM (0.320 mg/ml). If no agglutination was evident at this dilution, the plasma result was marked as >2.048 μM. One low-affinity OSP-specific IgG monoclonal antibody (B4) had agglutinating activity at 1.024 μM, whereas the other three monoclonal antibodies (G1, A4, and AT11) did not have detectable agglutinating activity at the highest concentration assessed (2.048 μM) (Table 1). Similar to our findings using polyclonal antibodies described above, we found that the anti-OSP monoclonal antibodies also blocked V. cholerae motility in a concentration-dependent manner, including at subagglutinating concentrations, whereas the anti-flagellin antibody did not block motility (Fig. 3) (*P* ≤ 0.001 to 0.05) (see Movies S1, S2, and S3). Inhibition of “shooting star” motility at the single bacterial cell level (without agglutination and clumping) was demonstrated by analysis of freeze-frame images of V. cholerae in the presence of human monoclonal OSP-specific antibodies but not in the presence of the anti-flagellin antibody (Fig. 3 and 4; Movies S1, S2, and S3). There were no apparent differences in the capacities of the high- versus low-affinity OSP-specific antibodies to inhibit V. cholerae motility (Fig. 3 and 4). Scanning electron microscope imaging of V. cholerae revealed that the OSP-specific monoclonal antibody promoted flagellar tethering, but tethering was occasionally observed in the presence of flagellum-specific monoclonal antibody (see Fig. S3). Occasional bacterial blebbing was also observed in scanning electron microscopic analyses with both antibodies. Our attempts to confirm the potential requirement for antibody mediated cross-linking using the monoclonal antibodies were confounded by aggregation of monoclonal Fab fragments under all assessed nonreducing conditions.

10.1128/mBio.02847-20.3 FIG S3 Scanning electron microscopy of V. cholerae mixed with monoclonal antibodies. Scanning electron microscope (SEM) photos taken using a Supra55VP microscope with a SE2 laser. (A and B) V. cholerae O1 O395 (OD of 0.1) and anti-flagellin monoclonal antibody (AT11 0.025 μM); magnification range, 45,300 to 46,700. (C and D) V. cholerae O1 O395 (OD of O.1) and anti-OSP monoclonal antibody (G1 0.005 μM); magnification range, 63,800 to 66,500. Download FIG S3, TIF file, 0.9 MB. Copyright © 2020 Charles et al. 2020 Charles et al. This content is distributed under the terms of the Creative Commons Attribution 4.0 International license.

10.1128/mBio.02847-20.7 MOVIE S1 V. cholerae motility after incubation with PBS. High-speed looped video microscopy after a 5-min incubation of V. cholerae O1 O395 (OD_600_ of 0.1) with phosphate-buffered saline. Note shooting star motility of individual V. cholerae. Download Movie S1, MOV file, 6.3 MB. Copyright © 2020 Charles et al. 2020 Charles et al. This content is distributed under the terms of the Creative Commons Attribution 4.0 International license.

10.1128/mBio.02847-20.8 MOVIE S2 V. cholerae motility after incubation with anti-FlaA monoclonal antibody. High-speed looped video microscopy after a 5-min incubation of V. cholerae O1 O395 (OD_600_ of 0.1) with a human monoclonal antibody targeting flagellin A (AT11; 0.25 μM). Note shooting star motility of individual V. cholerae. Download Movie S2, MOV file, 6.3 MB. Copyright © 2020 Charles et al. 2020 Charles et al. This content is distributed under the terms of the Creative Commons Attribution 4.0 International license.

10.1128/mBio.02847-20.9 MOVIE S3 V. cholerae motility after incubation with anti-OSP monoclonal antibody. High-speed looped video microscopy after a 5-min incubation of V. cholerae O1 O395 (OD_600_ of 0.1) with a human MAb targeting OSP (A4; 0.25 μM). Note significant diminution of shooting star motility of individual V. cholerae compared to what is observed following incubation with PBS (Movie S1) and anti-flagellin-specific monoclonal antibody (Movie S2). Download Movie S3, MOV file, 6.3 MB. Copyright © 2020 Charles et al. 2020 Charles et al. This content is distributed under the terms of the Creative Commons Attribution 4.0 International license.

### OSP-specific monoclonal antibody promotes survival and inhibits V. cholerae intestinal colonization in mice.

**FIG 5:**
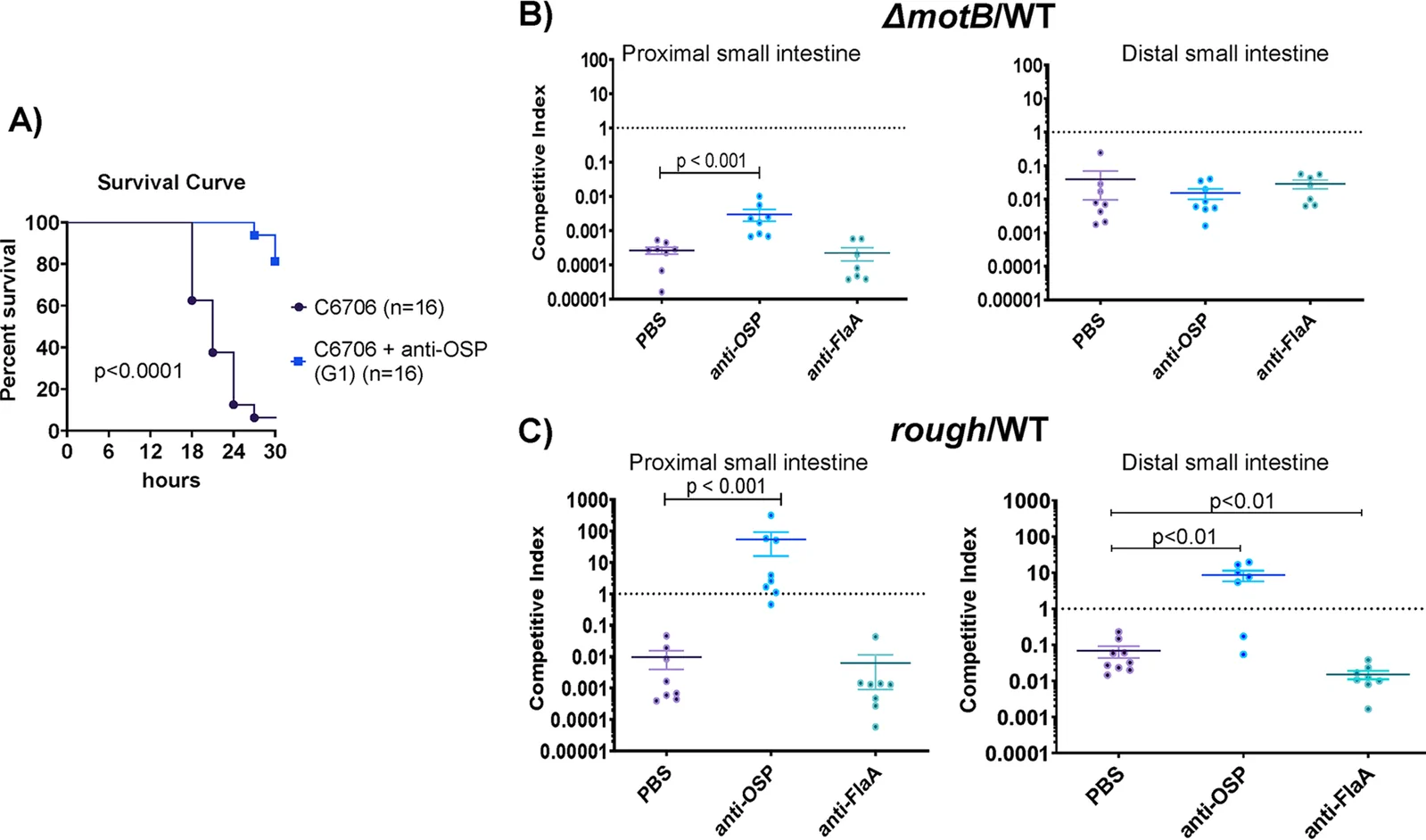
Impact of OSP-specific monoclonal antibodies on V. cholerae lethality and intestinal colonization in suckling mice. (A) OSP monoclonal antibody protects against death in the mouse neonatal V. cholerae challenge model. Wild type V. cholerae O1 strain C6706 (10^9^ organisms) was mixed with 0.25 μM human monoclonal antibody targeting V. cholerae OSP (G1) or PBS. Fifty microliters of sample mixture was inoculated into 3- to 5-day-old sucking CD-1 mice, and survival was assessed over a 30-h period every 3 h. Survival curves were compared by log rank testing. PBS, phosphate-buffered saline. (B and C) Inhibition of V. cholerae motility by OSP-specific monoclonal antibody prevents colonization of the proximal small intestine. V. cholerae strain C6706*lacZ*- (WT*LacZ*^−^) was competed against transposon mutants, i.e., nonmotile (*motB*::Kan^r^) (B) or rough (*VC0244*::Kan^r^) (C) strains, in 3- to 5-day-old suckling mice with the addition of PBS, human monoclonal antibody (MAb) targeting V. cholerae OSP (G1), or flagellin A (FlaA). The competitive index (CI) was calculated for the proximal third and distal third of the small intestine as the output ratio of mutant to WT*lacZ*^−^ strain divided by the input ratio of mutant to WT*lacZ*^−^. Each symbol represents the CI from a single mouse. Error bars represent the standard errors of the means. Differences between groups were assessed using a Mann-Whitney U test. PBS, phosphate-buffered saline; OSP, O-specific polysaccharide; FlaA, flagellin A.

We used a neonatal mouse challenge model to determine whether OSP-specific monoclonal antibody G1 alters the survival of suckling mice after lethal challenge with wild-type V. cholerae O1 strain C6706. Mixture of G1 with C6706 prior to orogastric inoculation led to a marked reduction in death of the mice (Fig. 5A) (*P* < 0.001). To assess how the effect of OSP-specific antibody on motility impacts V. cholerae survival and localization in the intestine, we carried out competition studies where the colonization of V. cholerae C6706*lacZ*^−^ (a strain that colonizes as well as the wild type) was compared to either (i) a C6706*lacZ*^+^ transposon mutant that was flagellated but nonmotile (*motB*::Kan^r^) or (ii) a C6706*lacZ*^+^ transposon mutant that was motile but rough and lacking OSP due to deficient perosamine synthesis (*VC0244*::Kan^r^). The motilities of the comparator and rough strains were equivalent, whereas the *motB*::Kan^r^ strain was confirmed as nonmotile. Relative to that for the C6706*lacZ*^−^ strain, the nonmotile *motB*::Kan^r^ mutant had a marked thousand-fold colonization defect in the proximal small intestine and a less pronounced 100-fold defect in the distal small intestine (Fig. 5B). When an OSP-specific monoclonal antibody was added to the inocula mixture at a subagglutinating concentration, the competitive disadvantage of the nonmotile mutant was significantly ameliorated in the proximal but not distal small intestine (Fig. 5B) (*P* < 0.001). This reduction in the competitive defect related to a decrease in the recovery of motile V. cholerae from the proximal small intestine in the presence of OSP-specific antibody G1 (see Table S3). In contrast, addition of the anti-flagellin monoclonal antibody to the inocula did not alter the competitive advantage of motile V. cholerae compared to that of the nonmotile mutant in either the proximal or distal intestine (Fig. 5B). These observations suggest that the inhibition of motility by the anti-OSP antibody impedes V. cholerae’s capacity to colonize the proximal small intestine.

In similar experiments where the comparator smooth C6706*lacZ*^−^ strain was competed with the rough (OSP^−^) mutant, we found that the rough strain had an ∼100-fold colonization defect in both the proximal and distal small intestine (Fig. 5C). Notably, recovery of the smooth comparator strain decreased in the presence of OSP-specific monoclonal antibody, in part abolishing the disadvantage of the rough strain in both the proximal and distal small intestine (Fig. 5C) (*P* < 0.001) (Table S3). In marked contrast, the anti-flagellin monoclonal antibody did not alter the competitive indices found in intestinal samples.

## DISCUSSION

We found that adult humans recovering from cholera develop antibody responses that inhibit V. cholerae motility. These antibodies, which impact motility at subagglutinating concentrations of antibody, target V. cholerae OSP and may mediate their effect via cross-linking. This work extends previous animal and *in vitro* LPS-based analyses (13–16).

V. cholerae motility is associated with pathogenesis (23). V. cholerae has a single sheathed polar flagellum and swims rapidly in an arcing pattern that can be visualized by dark-field microscopy of freshly passed cholera stools. This V. cholerae distinctive swimming pattern can be used to rapidly identify V. cholerae in the stool of potentially infected patients via dark-field microscopy and is referred to as “shooting star” motility (24). As shown in our videos (see Movies S1 to S3 in the supplemental material), the impact of the anti-OSP antibody on V. cholerae shooting star motility is significant. Motility is involved not only with horizontal movement of V. cholerae in the intestine (along the axis from stomach to colon) but also vertical localization in intestinal tissue (most importantly, along the axis created by villi and crypts in the small intestine) (25). Once ingested, the few V. cholerae cells that survive the acidic environment of the stomach enter the small intestine, and a subset swim into the lower villi and intestinal crypts (25). Motility is required for this vertical localization (25). V. cholerae motility is then involved in the initial penetration of intestinal mucin that overlays the intestinal epithelium (26). Following this initial penetration, V. cholerae cells become nonmotile (with many bacteria losing their flagella), transit the mucin (in a poorly understood process), adhere at the intestinal epithelial surface, where they form microcolonies, and secrete cholera toxin (26). This transition from a motile bacterium to an adherent microaggregated form at the intestinal surface is closely controlled by an interplay of quorum sensing by LuxO and HapR, motility, and V. cholerae virulence networks (26–33).

Following microcolony formation and the actions of cholera toxin on the intestinal epithelial cell leading to fluid secretion into the intestinal lumen, microcolonies break apart, and V. cholerae cells regain their motility in a poorly understood process. This “mucosal escape response” involves RpoS-dependent downregulation of virulence genes and upregulation of motility genes (34). V. cholerae motility facilitates the release of organisms from microcolonies (35). Detached organisms are then flushed/swim into the intestinal lumen and are passed into the environment in the rice water stools characteristic of cholera. These highly motile organisms have high transmission potential, a state that has been termed the “hyperinfectious state” (36, 37).

Based on this sequence of events, inhibition of V. cholerae mobility could impact V. cholerae pathogenesis at a number of critical points. Inhibition of motility could alter intestinal passage, reducing the number of bacteria that move horizontally through the intestinal tract. It could also impact the ability of V. cholerae to reach optimal ecologic niches in the lower third of intestinal villi and crypts and could affect the ability of V. cholerae to penetrate the protective mucin layer overlying the intestinal epithelium. Here, using the suckling mouse model, we found that the largest impact of anti-OSP antibodies was in the proximal small intestine, the region with the thickest mucin layer (25). Given the prominent interactions among the V. cholerae quorum sensing, motility, and virulence networks, the inhibition of motility by anti-OSP antibodies could modify the pathogen’s intestinal colonization, toxin expression, and virulence. Altered V. cholerae motility could also affect the ability of microcolonies to break apart and the ability of V. cholerae to reenter the intestinal lumen and be flushed from the patient, thus affecting the hyperinfectious and transmission potential of passed V. cholerae.

Our findings suggest that the effect of OSP-specific antibodies on V. cholerae motility requires at least bivalent binding of antibody. Fab fragments of polyclonal antibodies retained their capacity to bind OSP but did not impede V. cholerae motility. We also found that convalescent-phase IgM and IgA antibodies had a greater effect on V. cholerae motility than IgG; this may be secondary to the pentavalent and dimeric structures of IgM and IgA, respectively, which markedly increase their valency. Other groups have assessed the impact of murine antibodies on V. cholerae motility with variable results. Analysis of Fab fragments generated from polyclonal antibody generated in mice vaccinated against V. cholerae suggested that elimination of multivalency eliminated the effect of the antibodies on V. cholerae motility (16). However, Fab fragments generated from a number of murine monoclonal anti-LPS antibodies apparently affect V. cholerae motility (14, 15, 38).

Our observations may help explain this discrepancy. Despite clearly demonstrating complete cleavage of antibody and removal of the Fc (fragment, crystallizable) fraction, we were unable to prevent aggregation of Fab fragments of human monoclonal antibodies under any physiologic nonreducing condition at relevant concentrations. We did not observe aggregation in Fab fragments generated from human polyclonal antibodies, which were used in our analyses. The propensity of fragmented monoclonal antibodies to form aggregates, presumably due to their inability to form proper disulfide bonds, was described previously (39).

The requirement for multivalent binding to affect V. cholerae motility suggests at least two possible mechanisms for the OSP-specific effect on V. cholerae motility, depending on the concentrations of bacteria and antibody and the length of interaction time. The first requires sufficient bacteria, antibody, and time and involves interbacterial cross-linking of bacteria, i.e., agglutination. The second involves only the concentration of antibody and impact on individual bacteria. Our results strongly suggest that both conditions can impact V. cholerae motility, and in the human intestine, both conditions could exist. Not surprisingly, we were able to show an impact on V. cholerae motility under agglutinating conditions. More interestingly, we demonstrated the impact of human anti-OSP antibodies at antibody concentrations less than those associated with agglutination, and we were able to confirm inhibition of motility at the single bacterial level by microscopy. How anti-OSP antibodies could affect V. cholerae motility at subagglutinating concentrations is currently unclear. One possibility is flagellar tethering or kinking due to antibody-mediated OSP cross-linking (14). We did indeed detect flagellar tethering using electron microscopy, although we also observed occasional tethering with control antibody as well. Other mechanisms of action are also possible. For instance, the colonization defect of the flagellated but nonmotile *motB* mutant strain in comparison to that of the wild-type strain was not completely reversed when both were exposed to anti-OSP antibody, suggesting that the genetically nonmotile strain may be less able to respond to intestinal environmental signals and activate colonization and virulence cascades (40). An effect on transcriptional or protein expression in the setting of OSP-specific binding is also possible (41).

A limitation of our study was our inability to assess IgA and IgM fractions due to the small amounts recovered from polyclonal serum. V. cholerae is a noninvasive mucosal infection, and IgM and IgA are thought to be the predominant antibodies involved in mediating protection (11). We were, however, able to include analysis of monoclonal antibodies cloned from mucosa-homing plasmablasts in patients with cholera (22), and analyses comparing the impacts of IgM, IgA, and IgG isotype anti-OSP antibodies on V. cholerae motility are planned.

Despite this limitation, our results show that humans surviving cholera develop antibodies that inhibit V. cholerae motility through binding of the OSP component of LPS, under both subagglutinating and agglutinating conditions. These data build upon a growing body of evidence that OSP-specific antibody responses mediate protection against cholera and suggest that such antibodies may act by mechanisms other than agglutination. Finally, our findings also support a novel mechanism of antibody-mediated protection through direct inhibition of bacterial motility.

## MATERIALS AND METHODS

### Study subject selection and sample collection.

Venous blood was collected at the acute phase (day 2) and convalescent phase of infection (day 7) from 10 adult patients (age, 18 to 55 years) presenting to the International Centre for Diarrhoeal Disease Research, Bangladesh (icddr,b) hospital with culture-confirmed V. cholerae O1 El Tor Ogawa infection. Patients were treated with intravenous fluids and/or oral rehydration solution and antimicrobials at the discretion of the attending physician. This study was approved by the Research and Ethical Review Committees of the icddr,b and the human studies committee of Massachusetts General Hospital.

### Bacterial strains.

The V. cholerae O1 strains used in this study are listed in Table S2 in the supplemental material (42). Bacterial strains were grown at 37°C in Luria-Bertani (LB) medium with or without antibiotics as appropriate. Antibiotics and their concentrations were streptomycin (Sm) at a concentration of 100 μg/ml and kanamycin (Km) at 45 μg/ml. 5-Bromo-4-chloro-3-indolyl-β-d-galactopyranoside (X-Gal) (200 μg/ml) plus isopropyl β-d-1-thiogalactopyranoside (IPTG; 0.1 mM) were used for blue/white colony screening. For experiments with the rough strain, bacteria were resuspended in 0.5% NaCl to prevent autoagglutination after growth in LB medium.

10.1128/mBio.02847-20.5 TABLE S2 V. cholerae strains used in this study. Download Table S2, DOCX file, 0.1 MB. Copyright © 2020 Charles et al. 2020 Charles et al. This content is distributed under the terms of the Creative Commons Attribution 4.0 International license.

10.1128/mBio.02847-20.6 TABLE S3 V. cholerae recovery in competitive colonization assays. Download Table S3, DOCX file, 0.1 MB. Copyright © 2020 Charles et al. 2020 Charles et al. This content is distributed under the terms of the Creative Commons Attribution 4.0 International license.

### Purification of antibody isotypes from plasma.

IgG was purified from heat-inactivated human plasma using protein G HP spin trap columns (GE Healthcare) and then incubated with CaptureSelect IgA affinity matrix (Thermo Scientific) to remove contaminating IgA per the manufacturer’s instructions. IgA and IgM antibodies were then subsequently purified by sequentially running the flowthrough from the protein G column onto columns loaded with CaptureSelect IgA and IgM affinity matrix (Thermo Scientific). The quantity and purity of IgG, IgA, and IgM isotype fractions were determined by enzyme-linked immunosorbent assay (ELISA) using standard curves of ChromPure human IgG, IgA, and IgM (Jackson ImmunoResearch).

### Fab fragmentation of plasma IgG.

Fab fragments of IgG were generated using the Fab preparation kit (Pierce) per the manufacturer’s instructions. Briefly, immobilized papain was used to digest the desalted and purified IgG, and the resultant Fab fragments were purified using a Protein A Plus spin kit (Pierce). The purity of the Fab fragments was assessed by Western blotting with 100 ng of sample per well and direct probing with anti-IgG F(ab)_2_ and anti-IgG Fc conjugated to horseradish peroxidase (Jackson ImmunoResearch).

### Monoclonal antibody production.

Recombinant human monoclonal antibodies were generated from the cholera-induced day-7 plasmablast population of V. cholerae-infected Bangladeshi patients by single-cell expression as previously described (22). For this analysis, we used the following previously characterized human IgG monoclonal antibodies: high-affinity anti-OSP G1 (CF21.2.G01), low-affinity anti-OSP A4 (CF21.1.A04), low-affinity anti-OSP B4 (CF21.1.B04), and anti-FlaA-flagellin AT11 (AT11.1.B12) (22).

### Generation of V. cholerae O1 OSP.

OSP from V. cholerae O1 Ogawa strain PIC158 was purified and conjugated to bovine serum albumin (BSA) as previously described (43).

### Vibriocidal assays.

Vibriocidal assays were performed on heat-inactivated plasma samples as previously described (44), and the target strain V. cholerae O1 Ogawa O395 was used. Heat-inactivated plasma and exogenous guinea pig complement (EMD Millipore) were incubated with the target strain. Vibriocidal titers were defined as the reciprocal of the highest serum dilution resulting in a 50% reduction in optical density at 600 nm (OD_600_) compared to that of no serum controls.

### OSP ELISA.

OSP responses were measured using an ELISA as previously described (44). Microplates were coated with 100 ng/well of O1 Ogawa OSP-BSA. Samples were added to plates (plasma, 1:50; purified IgG, 10 μM; purified Fab, 1 μM), and IgG responses were detected with goat anti-human IgG conjugated to horseradish peroxidase [anti-IgG F(ab)_2_ for Fab] (Jackson ImmunoResearch). IgM and IgA anti-OSP reactivity of plasma was measured using goat anti-human IgM and IgA conjugated to horseradish peroxidase (Jackson ImmunoResearch). Peroxidase activity was measured with the substrate 2,2-azinobis (ethylbenzthiazolinesulfonic acid) for plasma, *o*-phenylenediamine for purified IgG/M/A, and SuperSignal West Femto maximum sensitivity substrate (Thermo Scientific) for purified Fab fragments.

### Agglutination assay.

Agglutination assays were performed as previously described with modification to match conditions used in assessing motility via high-speed microscopy (22). Briefly, V. cholerae O1 classical Ogawa strain O395 was grown to mid-log phase in bovine heart infusion medium. Bacteria were pelleted, washed twice with phosphate-buffered saline (PBS), and diluted to an OD_600_ of 0.1 with PBS. In a U-bottom microtiter plate blocked with BSA, 25 μl of bacterial samples was mixed with equal volumes of day-7 patient samples to a final starting dilution/concentration of plasma 1:8; purified IgG at 2.5, 1, or 0.5 μM depending on sample availability, purified IgG Fab at 1 μM, or monoclonal antibody starting at a concentration of 2.048 μM (0.320 mg/ml). The minimum agglutinating titer was determined by serial 2-fold dilutions of antibody sample. Plates were incubated at room temperature for 5 min and imaged using a UV imaging system (ChemiDoc; Bio-Rad) to assess agglutination. If no agglutination was evident, the value was marked as greater than the most concentrated value.

### Viability assay.

V. cholerae O1 classical Ogawa strain O395 was grown to mid-log phase in LB medium. Bacterial samples, adjusted to an OD_600_ of 0.1, were added to equal volumes of PBS or patient’s sample (heat-inactivated convalescent plasma or purified IgG for final antibody concentration of 1:256 for plasma and 0.25 μM and 2.5 μM for purified IgG). The mixture was incubated for 5 min at 37°C. The effect of the antibody on bacterial viability was determined by serially diluting samples 10-fold and enumerating bacteria on LB plates. Experiments were performed in quadruplicates.

### Motility inhibition assay.

V. cholerae motility was assessed using a modification of a previously reported approach (13). Briefly, V. cholerae O1 O395 were grown to mid-log-phase in LB broth, and the OD_600_ was adjusted to 0.1. This OD was found to approximate 3 × 10^6^ CFU/ml. Heat-inactivated convalescence antibodies were mixed with bacterial sample at a 1:1 dilution of acute or convalescence antibodies (final antibody concentrations of plasma and purified IgG were at subagglutinating levels: 1:256 (plasma), 0.25 μM [polyclonal IgG], and 0.5 μM [Fab generated from purified polyclonal IgG]), and 10 μl of the mixture was placed on a standard glass slide. Concentration dependence was also assessed for purified polyclonal IgG and monoclonal antibodies using 10-fold serial dilutions (2.5 to 0.025 μM for purified IgG; 2.5 to 0.00025 μM for monoclonal antibodies) using slides blocked with BSA. Motility inhibition of pooled samples of IgM and IgA was assessed at 0.25 μM; sample volumes were too low to assess motility individually for each patient using these antibody isotypes. PBS mixed 1:1 with bacteria was used as a negative control. Slides were incubated for 5 min at 37°C before visualization of motility by dark-field microscopy (Nikon Eclipse Ti-E inverted microscope stand, Nikon Plan-Fluor 40×/0.75 lens objective, Nikon TI-DF dark-field dry condenser [numerical aperture, 0.95 to 0.80], Hamamatsu ORCA-ER camera, and MetaMorph imaging software). Images were taken at 400-ms exposure time over 100 frames with readout time frame of 111.72 ms (movie play time of 1/30th s per frame). Frames 1, 50, and 98 were frozen as still shots, and bacteria were counted as lines (motile bacteria) or dots (nonmotile bacteria). Results were expressed as percent motile (motile/total bacteria) and averaged across the three screen shots. To confirm that the antimotility effect was secondary to OSP-specific antibodies, we performed experiments with antibody samples that were first adsorbed with OSP-BSA. Antibody samples were first incubated overnight with OSP-BSA before mixing with the bacterial sample for microscopy. Adsorption was performed using a 1:100 molar ratio (antibody/OSP) for purified antibody, and 250 μg of OSP-BSA for diluted plasma samples. PBS mixed with OSP-BSA was used as a negative control. All assays were performed in at least triplicates.

### Electron microscopy.

Scanning electron microscopy (SEM) was performed at the Harvard University Center for Nanoscale Systems (CNS) using a FESEM Supra55VP microscope with an SE2 laser. V. cholerae O1 O395 was imaged in the presence or absence of anti-flagellin monoclonal antibody (AT11, 0.025 μM) at a magnification range of 45,300 to 46,700 or anti-OSP monoclonal antibody (G1, 0.005 μM) at a magnification range of 63,800 to 66,500.

### Neonatal challenge.

The mouse neonatal V. cholerae challenge assay was used to assess protection afforded by OSP-specific antibodies as previously described (6). Briefly, 3- to 5-day-old CD-1 mice were separated from their dams for 3 h and then orally inoculated with 50 μl of a mixture containing 10^9^ V. cholerae O1 Inaba C6706 and PBS or anti-OSP monoclonal antibody (G1) at a final concentration of 0.25 μM. This strain was used due to the availability of mutant strains used in the competition assays (see below). Mice were then housed away from the dams at 30°C and monitored for survival for 30 h. All mouse studies were approved by the MGH Institutional Animal Care and Use Committee.

### Competitive colonization assay.

A single colony of each V. cholerae strain was inoculated into LB medium with Sm for C6706*lacZ*^−^ (referred to below as wild type [WT]) and LB medium with Sm and Kan for C6706*lacZ^+^* transposon mutants (rough [*VC0244*::Kan^r^] and nonmotile [*motB*::Kan^r^]) and incubated at 37°C overnight on a roller drum. Cultures were then regrown to mid-log phase, and the rough and comparator wild-type (WT) strains were mixed together 1:1 (10^5^ organisms) and resuspended in 50 μl of LB medium without antibiotic mixed with PBS or 0.025 μM monoclonal antibody G1. A higher inoculum of the nonmotile strain was required to enable sufficient recovery after infection for enumeration and calculation of a competitive index (input, 10^6^ mutant and 10^4^ comparator). Three- to 5-day-old CD-1 infant mice were inoculated by oral gavage. After 20 h, the pups were sacrificed, and the proximal and distal thirds of the small intestines were isolated and mechanically homogenized in 5 ml of LB medium. Serial dilutions were plated on LB with Sm, X-Gal, and IPTG to enumerate bacteria and determine the input and output ratios of the comparator WT and competing rough or nonmotile strains. The competitive index was calculated as the output ratio of competing strain/WT strain divided by the input ratio of competing strain/WT. The data represent infections of multiple litters on different days that were pooled for analysis. All mouse studies were approved by the MGH Institutional Animal Care and Use Committee.

### Statistical analysis.

Results were expressed as medians and compared by a Wilcoxon matched-pairs signed-rank sums test or Mann-Whitney U test for within group and between group comparisons as appropriate. Results of the survival curve analysis were compared by log rank testing. The threshold for statistical significance was a two-tailed *P* value of <0.05. Prism 6 was used for all statistical analyses.
