# Supplementary material for: Humans Surviving Cholera Develop Antibodies against Vibrio cholerae O-Specific Polysaccharide That Inhibit Pathogen Motility
Source: mBio. 2020 Nov 17;11(6):e02847-20. doi: 10.1128/mBio.02847-20 (PMC7683404; doi:10.1128/mBio.02847-20)
Supplement: TABLE S1 [file mBio.02847-20-st001.docx]

**Table S1:** **Cholera patient characteristics for polyclonal sera. All were infected with *V. cholerae* O1 Ogawa in Dhaka, Bangladesh.**

| Patient | Age (yrs) | Sex | ABO blood group | Vibriocidal titer D2* | Vibriocidal  titer D7* |
| --- | --- | --- | --- | --- | --- |
| 1 | 51 | F | O | 0 | 1280 |
| 2 | 32 | M | AB | 0 | 1280 |
| 3 | 25 | M | A | 2 | 1280 |
| 4 | 26 | F | B | 0 | 1280 |
| 5 | 35 | M | B | 10 | 160 |
| 6 | 44 | F | B | 0 | 160 |
| 7 | 24 | F | A | 10 | 640 |
| 8 | 50 | M | O | 0 | >10240 |
| 9 | 40 | M | O | 0 | 640 |
| 10 | 40 | M | A | 0 | 320 |

*Vibriocidal titer to *V. cholerae* O1 classical Ogawa strain O395 defined as the reciprocal of the highest plasma dilution resulting in greater than 50% reduction in optical density (i.e. cholera growth) when compared to control wells without plasma
