# Supplementary figures and images for: Humans Surviving Cholera Develop Antibodies against Vibrio cholerae O-Specific Polysaccharide That Inhibit Pathogen Motility
Source: mBio. 2020 Nov 17;11(6):e02847-20. doi: 10.1128/mBio.02847-20 (PMC7683404; doi:10.1128/mBio.02847-20)

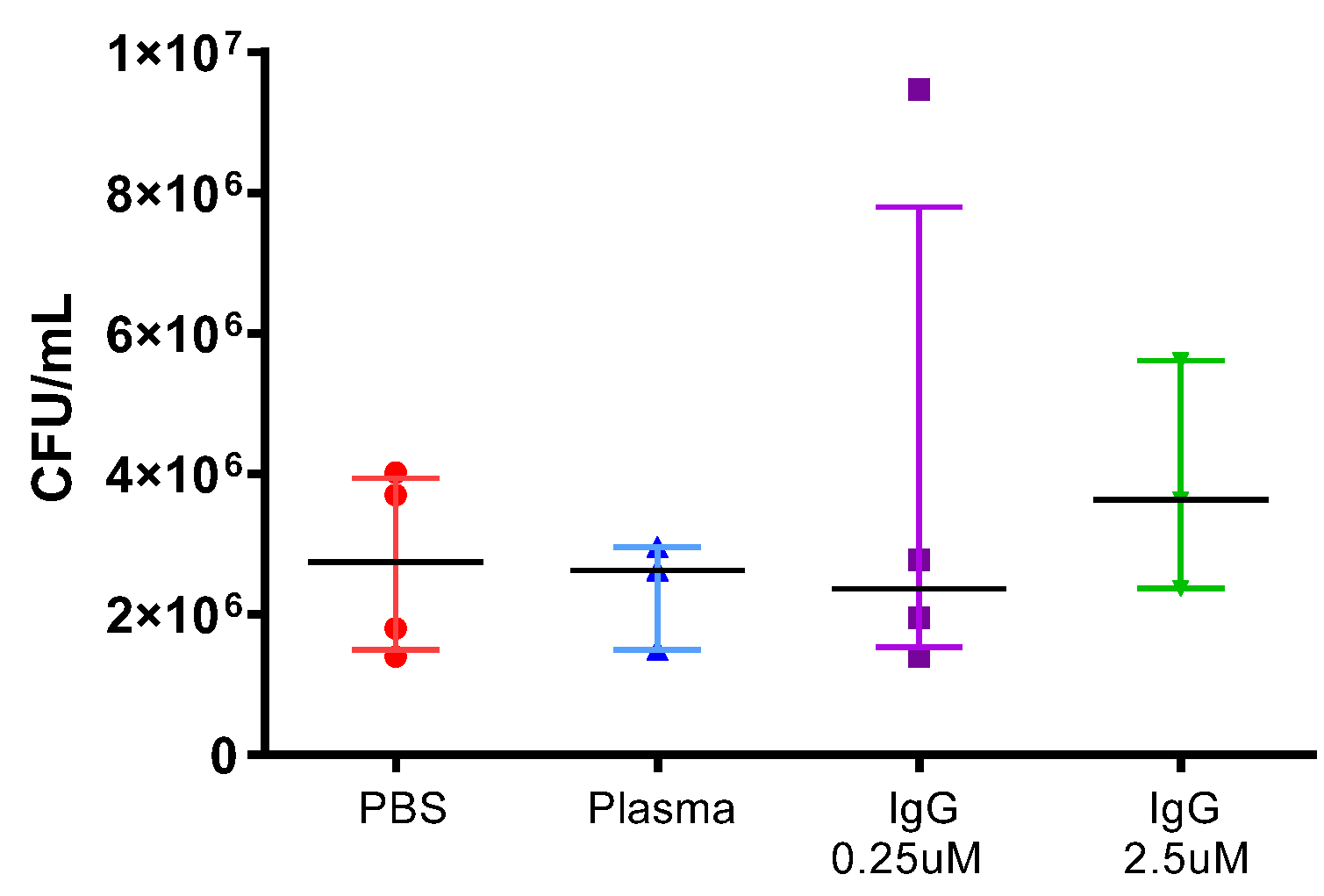

Supplement: FIG S1 [file mBio.02847-20-sf001.tif]

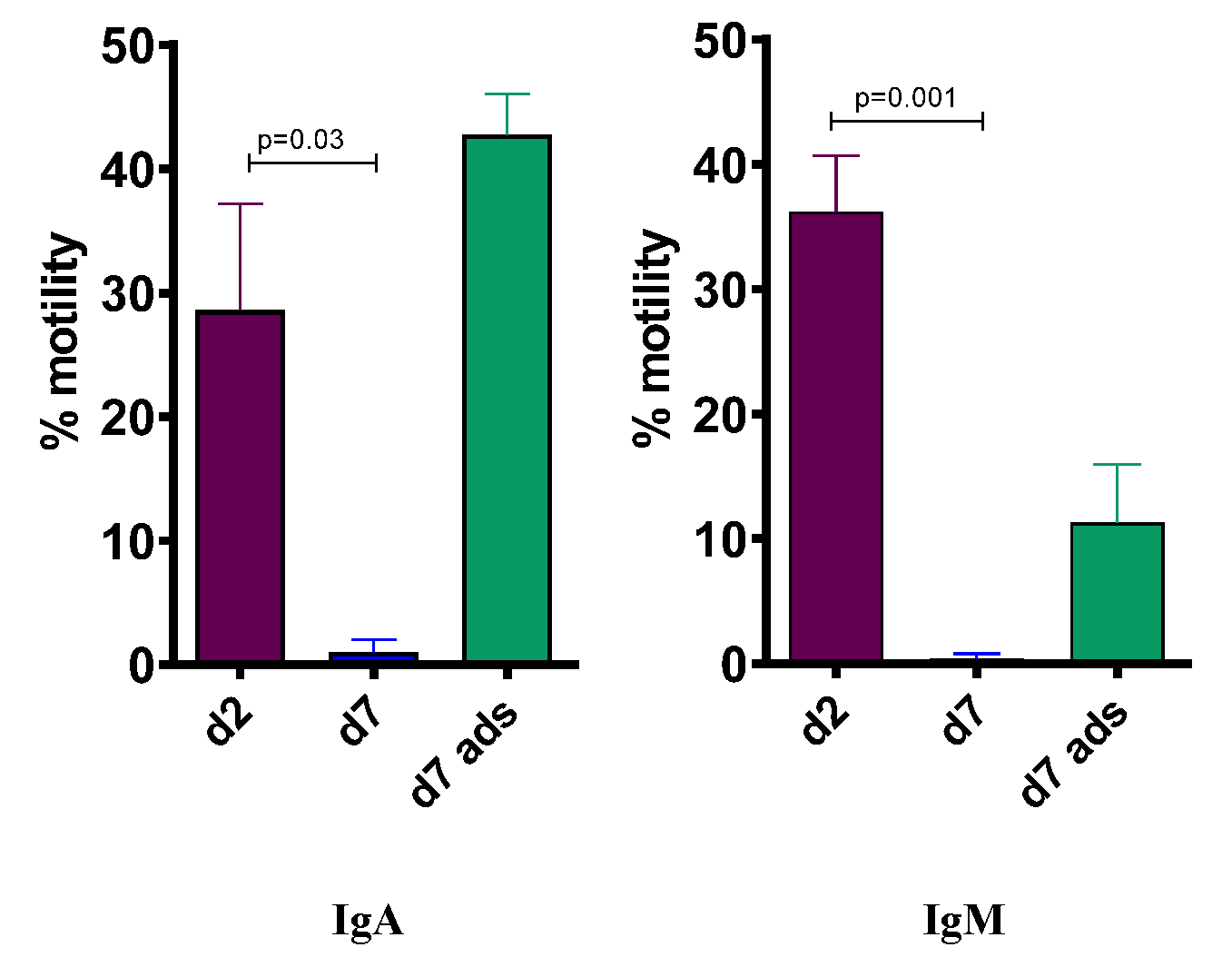

Supplement: FIG S2 [file mBio.02847-20-sf002.tif]

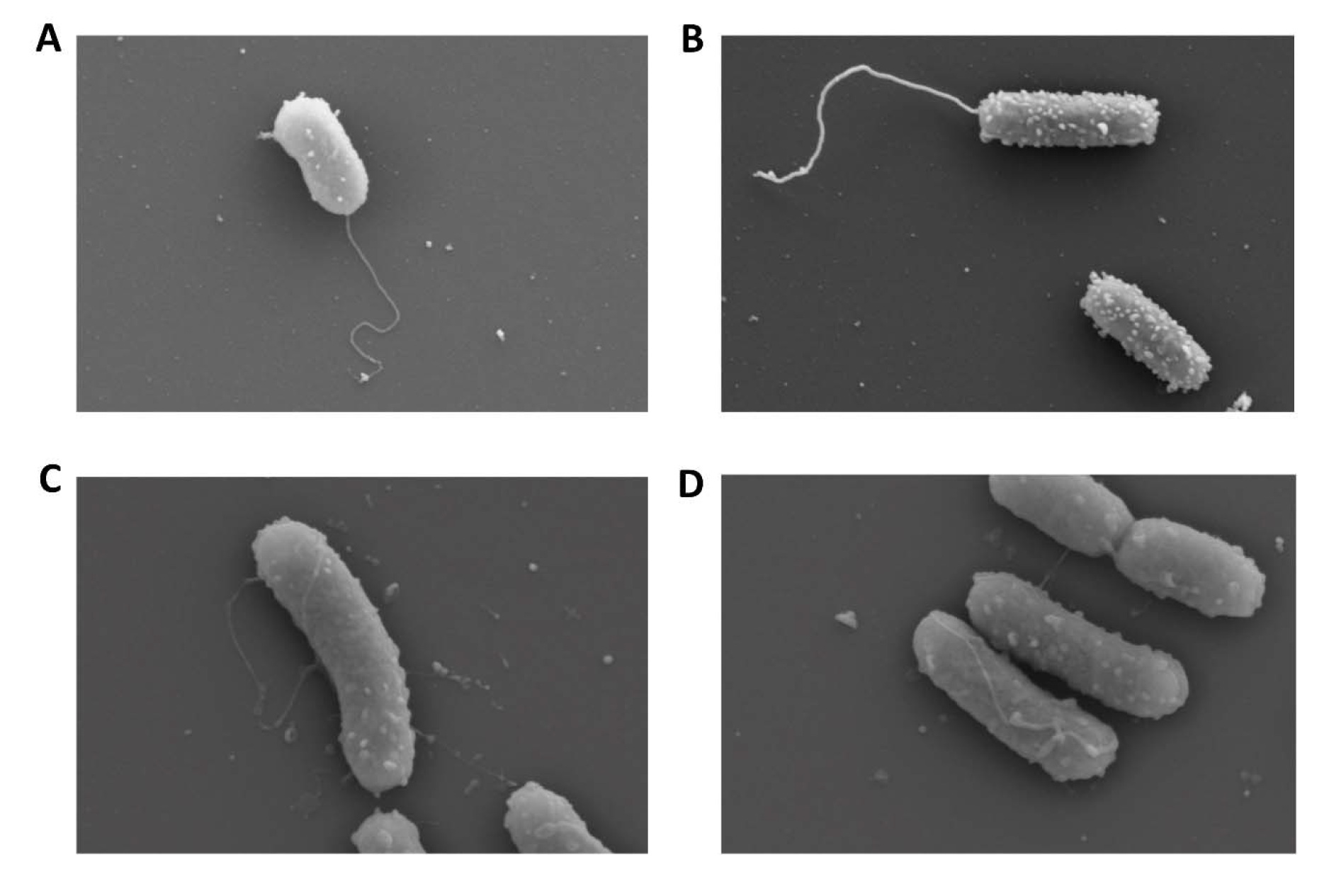

Supplement: FIG S3 [file mBio.02847-20-sf003.tif]
