# Supplementary material for: Humans Surviving Cholera Develop Antibodies against Vibrio cholerae O-Specific Polysaccharide That Inhibit Pathogen Motility
Source: mBio. 2020 Nov 17;11(6):e02847-20. doi: 10.1128/mBio.02847-20 (PMC7683404; doi:10.1128/mBio.02847-20)
Supplement: TABLE S2 [file mBio.02847-20-st002.docx]

**Table S2:** ***V. cholerae* strains used in this study**

| *V. cholerae* strains | Genotype and description | Assay | Reference and source |
| --- | --- | --- | --- |
| Wild type *V. cholerae* O1 strains | | | |
| O395 | Classical Ogawa strain | Motility video-microscopy; vibriocidal, viability, agglutination assays |  |
| C6706 | El Tor Inaba Strain | Neonatal *V. cholerae* challenge; MD50 |  |
| Mutants of C6706 | O1 El Tor Inaba Strain |  |  |
| *LacZ^-^*, Sm^r^ | El Tor Inaba Strain Sm-resistant; spontaneous LacZ^-^ |  | (34) |
| *motB::*Kan^r^ | Sm-resistant; flagellated but non-motile strain | Competitive index | (34) |
| *VC0244*::Kan^r^ | Sm-resistant; motile but rough strain | Competitive index | (34) |

**Sm: streptomycin; Kn: kanamycin**
