# Supplementary material for: Humans Surviving Cholera Develop Antibodies against Vibrio cholerae O-Specific Polysaccharide That Inhibit Pathogen Motility
Source: mBio. 2020 Nov 17;11(6):e02847-20. doi: 10.1128/mBio.02847-20 (PMC7683404; doi:10.1128/mBio.02847-20)
Supplement: TABLE S3 [file mBio.02847-20-st003.docx]

Table S3. *V. cholerae* recovery in competitive index assays

| **Motility mutant/comparator strain: proximal small intestine** | | | |  | **Motility mutant/comparator strain: distal small intestine** | | | |
| --- | --- | --- | --- | --- | --- | --- | --- | --- |
|  | MotB mutant (LacZ+) | Wild type (LacZ-) | CI |  |  | MotB mutant (LacZ+) | Wild type (LacZ-) | CI |
| **PBS** | 2153 | 723854 | 0.00018 |  | PBS | 1429395 | 6445250 | 0.01 |
| **Anti-OSP** | 2104 | 66540 | 0.00192 |  | Anti-OSP | 211122 | 1007941 | 0.009 |
| **Anti-FlaA** | 2857 | 1448837 | 0.00012 |  | Anti-FlaA | 2599605 | 5788389 | 0.020 |
|  |  |  |  |  |  |  |  |  |
| **Rough mutant/comparator strain: proximal small intestine** | | | |  | Rough mutant/comparator strain: distal small intestine | | | |
|  | MotB mutant (LacZ+) | Wild type (LacZ-) | CI |  |  | MotB mutant (LacZ+) | Wild type (LacZ-) | CI |
| **PBS** | 1556 | 278222 | 0.002 |  | PBS | 9737081 | 11787820 | 0.044 |
| **Anti-OSP** | 100288 | 5765 | 7.23 |  | Anti-OSP | 3348823 | 65498 | 2.77 |
| **Anti-FlaA** | 1443 | 589650 | 0.001 |  | Anti-FlaA | 275680 | 1320070 | 0.011 |

Geometric mean quantitative colony forming unit counts from 3 cms of proximal or 3 cms of distal small intestine as described in text. Competitive indices are calculated as mutant strain/comparator strain divided by input ratio. MotB mutant: C6706*lacZ*^+^ transposon mutant that is flagellated but non-motile (*motB*::Kan^r^); Rough mutant: C6706*lacZ*^+^ transposon mutant that is rough and lacking OSP (*VC0244-*perosamine synthase::Kan^r^); Comparator strain: *V. cholerae* strain C6706*lacZ*^-^. PBS, Phosphate buffered saline; anti-OSP, monoclonal IgG OSP-specific antibody G1; anti-FlaA, monoclonal IgG Flagellin A-specific antibody AT11.
